# Tocilizumab modulates the activity of the classical and alternative complement pathways in rheumatoid arthritis patients

**DOI:** 10.3389/fimmu.2025.1486588

**Published:** 2025-01-30

**Authors:** Iván Ferraz-Amaro, Sergio Santos-Concepción, Javier Castro-Hernández, Maria Vanesa Hernández-Hernández, Beatriz Tejera Segura, Cristina Luna, Esmeralda Delgado-Frias, Federico Díaz-González

**Affiliations:** ^1^ Servicio de Reumatología, Hospital Universitario de Canarias, San Cristóbal de La Laguna, Spain; ^2^ Departamento de Medicina Interna, Dermatología y Psiquiatría, Universidad de La Laguna (ULL), San Cristóbal de La Laguna, Spain; ^3^ Departamento de Medicina Física y Farmacología, Área de Farmacología Universidad de La Laguna (ULL), San Cristóbal de La Laguna, Spain; ^4^ Servicio de Reumatología, Hospital Universitario Insular-Materno infantil de Canarias, Las Palmas de Gran Canarias, Spain; ^5^ Servicio de Reumatología, Hospital Nuestra Señora de la Candelaria, Santa Cruz de Tenerife, Spain; ^6^ Instituto Universitario de Tecnología Biomédica (ITB), Universidad de La Laguna, San Cristóbal de La Laguna, Spain

**Keywords:** tocilizumab, rheumatoid arthritis, disease activity, classical complement system activity, alternative complement system activity

## Abstract

**Background:**

Tocilizumab (TCZ) is a monoclonal antibody that neutralizes interleukin (IL)-6 and is indicated for diseases characterized by markedly elevated inflammatory markers, such as rheumatoid arthritis (RA). The complement system has been implicated in the etiopathogenesis of RA.

**Objective:**

To evaluate the effect of systemic IL-6 inhibition on complement pathways functional activity in RA patients treated with TCZ.

**Desing:**

Prospective non-interventional study.

**Methods:**

Twenty-seven RA patients included in the TOCRIVAR study who received TCZ (8mg/kg IV/q4w) were evaluated at baseline and at weeks 12, 24 and 52 of treatment. Disease activity, as assessed by composite indices, acute phase reactants, and new-generation functional assays of the three complement pathways, was evaluated at baseline and at each follow-up visit. Multivariable linear mixed models were used to determine changes in the complement system cascades over time.

**Results:**

After adjustment for disease activity, basal levels of the classical and alternative pathways decreased significantly after TCZ treatment. The effect on the classical pathway remained significant after 52 weeks. The decrease in the alternative pathway was significant at weeks 12 and 24, but not at week 52 of TCZ treatment. TCZ had no effect on the lectin cascade throughout the follow-up.

**Conclusion:**

TCZ reduces the activity of the classical and alternative pathways of the complement system in RA patients regardless of the improvement in disease activity. This finding may contribute to a better understanding of the mechanisms by which the IL-6 blockade reduces disease activity in RA patients.

## 1 Introduction

Tocilizumab (TCZ) is a monoclonal antibody that neutralizes interleukin (IL)-6 signaling by binding to the soluble and membrane-bound forms of the human IL-6 receptor. TCZ is indicated for rheumatoid arthritis (RA) (1), juvenile idiopathic arthritis (2), giant cell arteritis (3) patients, and has also shown benefits in severe cytokine release syndrome (4). In addition, there is also robust evidence from randomized trials supporting the use of TCZ in the treatment of patients with COVID-19 (5, 6).

IL-6 is a pleiotropic cytokine that plays a critical role in the immune response, including effects on B and T lymphocytes, synovial inflammation, and hematopoiesis (7). In addition, IL-6 is a potent inducer of acute phase reactants, proteins involved in the coagulation cascade and several proteins of the complement system (8, 9). Consequently, IL-6 is a cytokine that plays a pivotal role in the activation and regulation of the immune response, including the complement system (7, 10, 11).

The complement system is an essential component of the innate immune response, functioning alongside antibody-mediated mechanisms. It consists of approximately 60 plasma and cell membrane proteins organized into three interconnected activation pathways: the classical, alternative, and lectin pathways. All modes of complement activation converge on the proteolysis of C3 and C5, generating the potent pro-inflammatory peptides C3a and C5a, while C5b initiates the membrane attack complex (MAC) formation (C5b-9), which is responsible for direct cell lysis (12), The complement system has been linked to the etiopathogenesis of RA. The formation of immune complexes in inflamed synovial tissue (pannus) and cartilage has been shown to trigger complement activation and consumption of complement components (13). Activation products such as the C1 inhibitor-C1r-C1s complexes C2a, C3a, C3d or C3dg, and C5a have been observed at elevated levels in synovial fluid (14), and complement deposition can be visualized in synovial tissue through immunohistochemical staining (15). Moreover, the functional CH50 test has been described as elevated in RA patients compared to controls (16, 17). In contrast to other immune-mediated diseases such as systemic lupus erythematosus (SLE), RA disease activity is not characterized by complement consumption.

In this study, we used new-generation assays to assess the functional activity of three pathways of the complement system. Our aim was to analyze whether systemic IL-6 blockade affects the activity levels of these complement pathways. To address this question, a cohort of RA patients with active disease who prescribed TCZ under standard clinical practice conditions were followed for one year. Serial measurements of the functional activity levels of the three complement pathways were performed throughout the study period.

## 2 Patients and methods

### 2.1 Study participants

TOCRIVAR (Effect of the Monoclonal Anti-IL6 Antibody -Tocilizumab- on Cardiovascular Risk in Patients with Rheumatoid Arthritis) (ClinicalTrials.gov NCT017523359) is a one-year prospective study that examined the impact of TCZ on cardiovascular risk factors in RA patients. Twenty-seven RA patients were recruited for the study. All patients were diagnosed in accordance with the 2010 ACR/EULAR classification criteria for RA (18) for a period ≥6 months before inclusion. Inclusion criteria included the following: a diagnosis of active moderate to severe RA (≥3.2 disease activity score -DAS- in 28 joints); patients who demonstrated an inadequate clinical response to a stable dose of non-biological disease-modifying antirheumatic drugs (DMARD) or who had failed to respond to one anti-tumor necrosis factor (TNF) treatment for a period ≥8 weeks prior to treatment; and those patients who were receiving oral glucocorticoids, the dose of which should have been ≤10 mg prednisone or equivalents and who remained at a stable dose for at least one month prior to the start of TCZ. All patients were offered open-label treatment with TCZ 8 mg/kg every 4 weeks for 12 months between January 2012 and July 2015. Open-label assessments were performed at baseline and at weeks 12, 24, and 52. Patients diagnosed with any other rheumatic disease, hepatitis C infection, active diverticulitis, latent tuberculosis (based on positive PPD or an interferon gamma release assay or suspicious chest X-ray), or who were pregnant, or lactating were excluded.

This trial was approved by an independent ethics committee and an institutional review committee of the Hospital Universitario de Canarias (Spain) and was assigned the reference number FRC-TOC-2009-01 (TOCRIVAR). All patients who participated in the study provided written informed consent.

### 2.2 Data collection

The subjects completed a cardiovascular risk factor and medication use questionnaire and underwent a physical examination at every visit. Weight, height, body-mass index (BMI), waist-to-hip ratio and systolic and diastolic blood pressure (measured with the participant in a supine position) were assessed under standardized conditions. Information regarding smoking status (current smoker versus non-smoker), diabetes, and hypertension was obtained from the questionnaire. At every visit, disease activity, disability and physical activity were assessed. Disease activity was measured using the DAS28, with the form that addresses both erythrocyte sedimentation rate (-ESR) and C-reactive protein (-CRP) (19), the Clinical Disease Activity Index (CDAI) (20) and the Simple Disease Activity Index (SDAI) (20). Disease disability was determined using the Health Assessment Questionnaire (HAQ) (21).

### 2.3 Complement assessments

The SVAR functional complement assays (Wieslab^®^ brand, Sweden) were used to assess classical, alternative and lectin pathway activity. These tests combine principles from the hemolytic assay for complement function with the use of labeled antibodies that specifically target the neoantigen produced because of complement activation. The quantity of neoantigen generated is directly proportional to the functional activity of the complement pathways. Briefly, patient serum stored at -80°C was thawed and diluted with a specific blocking agent to ensure activation of only the complement pathway of interest. The serum was then incubated in wells coated with specific activators of each of the three complement pathways. After well washing, the presence of C5b-C9 (membrane attack complex or MAC) was measured by absorbance (optical density) using an alkaline phosphatase-labeled antibody specific for the neoantigen expressed during its formation. Results are expressed qualitatively for the alternative and lectin pathways using a negative and positive control, and semi-quantitatively for the classical pathway using a calibration curve, following the manufacturer’s instructions. In these assays, lower levels indicate a diminished functional activity of the complement pathways under investigation. This may be attributed to either consumption by activation or a reduction in production of the proteins involved in the cascade. Wieslab^®^ has validated these functional assays by studying their correlation and concordance with the classical CH50 and AH50 hemolytic tests (https://www.svarlifescience.com/).

### 2.4 Statistical analysis

Sample size was estimated based on the anticipated effect size and intraclass correlation, using a simulation-based approach appropriate for mixed-effects models to achieve 80% power at a 5% significance level. This method allowed for adjustments based on the longitudinal design and potential within-subject correlations. Accordingly, we estimated that we would need to enroll 24 patients. Demographic and clinical characteristics in patients with RA at baseline and in each visit are described as mean (standard deviation, SD) or percentages for categorical variables. For non-normally distributed continuous variables, data are expressed as median and interquartile ranges (IQR). To avoid any impact on the statistical analyses of data lost during the follow-up period, variations in clinical characteristics, disease activity scores, acute phase reactants, and complement system route assays were analyzed using multivariable linear regression mixed models for repeated measures. Since changes in complement functional tests after TCZ could also be influenced by improvement in disease activity, a multivariable analysis adjusted for CDAI was also performed. Thus, the evolution of complement pathways was analyzed both univariably and adjusting for this score to illustrate whether the effect of TCZ was independent of changes that could have occurred due to improvement in disease activity. These analyses were performed using mixed models. Mixed models offer several advantages over paired data analyses because they can handle missing data more effectively, accommodate unbalanced designs, and account for both fixed and random effects. All tests were two sided with a P < 0.05 significance level using Stata software, version 17/BE (StataCorp, College Station, TX, USA). Graphs were generated using GraphPad Prism 10 version 10.2.3, GraphPad Software, San Diego, California, USA, www.graphpad.com.

## 3 Results

### 3.1 Characteristics of the participants and changes in RA disease activity during one year of follow-up

A total of 27 RA patients, consisting of 24 females and 3 males with a mean age of 52 ± 11 years, were included in this study. **Table 1** shows the demographic data, disease characteristics, and comorbidities of the participants when they started treatment with TCZ. The median disease duration was 8 years (IQR 2–12), and 69% tested positive for both anti-citrullinated protein antibodies (ACPA) and rheumatoid factor. The patients exhibited moderate-severe RA disease activity as indicated by DAS28-ESR (5.77 ± 0.88), SDAI (29 ± 10), and CDAI (27 ± 10) scores. Twenty patients (77%) were on prednisone, and 19 (70%) were receiving TCZ in combination with either methotrexate or leflunomide. Only 8 patients (30%) were on TCZ monotherapy. Those on prednisone, methotrexate, or leflunomide maintained stable doses throughout the follow-up year.

**Table 1:** Baseline demographic, comorbidities, and disease-related characteristics of RA patients.

	Patients (n=27)
Female, n (%)	24 (92)
Age, years	52 ± 11
Weight, kg	71 ± 14
Height, cm	160 ± 7
BMI, mg/cm2	28 ± 5
Waist circumference, cm	95 ± 14
Hip circumference, cm	102 ± 14
Waist/hip ratio	0.93 ± 0.05
Systolic pressure, mmHg	133 ± 23
Diastolic pressure, mmHg	84 ± 9
Comorbidities	
Hypertension, n (%)	9 (35)
Diabetes, n (%)	4 (15)
Dyslipidemia, n (%)	12 (46)
Current smoker, n (%)	5 (19)
Anti-hypertension treatment, n (%)	9 (35)
Disease-related data	
Disease duration, years	8 (2-12)
Rheumatoid factor +, n (%)	18 (69)
ACPA +, n (%)	18 (69)
ESR, mm/h	40 (22-56)
CRP, mg/dl	8.8 (2.9-16.2)
Number of tender joints	10 (6-15)
Number of swollen joints	4 (2-7)
DAS28-ESR	5.77 ± 0.88
DAS28-CRP	5.16 ± 1.04
SDAI	29 ± 10
CDAI	27 ± 10
HAQ	1.250 (1.000-2.125)
Treatments	
Prednisone, mg/day	6 (2-8)
Current csDMARD	18 (69)
Methotrexate, n (%)	14 (54)
Leflunomide, n (%)	5 (19)

Data represent mean ± SD or median (interquartile rate).

ACPA, anti-cyclic citrullinated peptide antibody; BMI, body mass index; CDAI, Clinical Disease Activity Index; CRP, C reactive protein; csDMARD, conventional synthetic DMARD; DAS28, Disease Activation Score using 28 joints; DMARD, Disease-modifying antirheumatic drugs; ESR, erythrocyte sedimentation rate; HAQ, Health Assessment Questionnaire; SDAI, Simple Disease Activity Index; TNF, tumor necrosis factor.

BMI and waist circumference remained stable and did not reveal any differences after one year of treatment compared to baseline values. As expected, acute phase reactants and disease activity scores, including CDAI, significantly improved during the follow-up period (**Table 2**).

**Table 2:** Changes during treatment in terms of anthropometric characteristics and RA disease activity.

	Baseline	Week 12	p^1^	Week 24	p^2^	Week 52	p^3^
Anthropometrics							
BMI, kg/m2	28 ± 5	28 ± 5	0.41	28 ± 5	0.47	29 ± 6	0.085
Waist circumference, cm	95 ± 14	96 ± 13	0.85	96 ± 12	0.75	99 ± 12	0.83
Hip/waist circumference	0.93 ± 0.05	0.92 ± 0.05	0.34	0.92 ± 0.05	0.051	0.92 ± 0.05	0.091
RA disease activity							
ESR, mm/h	40 (22-56)	9 (5-16)	**<0.001**	10 (7-17)	**<0.001**	9 (6-24)	**<0.001**
CRP, mg/dl	8.8 (2.9-16.2)	1.1 (0.7-4.3)	**<0.001**	0.6 (0.5-1.1)	**<0.001**	1.5 (0.7-15.6)	**0.030**
Number of tender joints	10 (6-15)	3 (1-9)	**0.002**	3 (0-7)	**<0.001**	3 (0-11)	**0.007**
Number of swollen joints	4 (2-7)	1 (0-3)	**0.002**	0 (0-2)	**<0.001**	0 (0-4)	**0.005**
DAS28-ESR	5.77 ± 0.88	3.69 ± 1.20	**<0.001**	3.28 ± 1.33	**<0.001**	3.67 ± 1.69	**<0.001**
DAS28-CRP	5.16 ± 1.04	3.23 ± 1.19	**<0.001**	2.92 ± 1.12	**<0.001**	3.23 ± 1.80	**<0.001**
SDAI	29 ± 10	15 ± 10	**<0.001**	13 ± 9	**<0.001**	16 ± 13	**<0.001**
CDAI	27 ± 10	16 ± 10	**<0.001**	14 ± 9	**<0.001**	15 ± 12	**<0.001**
HAQ	1.250 (1.000-2.125)	1.000 (0.500-1.875)	0.061	1.063 (0.500-1.663)	**0.009**	1.250 (0.625-1.625)	0.36

Data represent mean ± SD or median (interquartile rate) at baseline and in every visit. The p-values at each visit reflect differences at weeks 12, 24, and 52 compared to baseline, using mixed models for continuous responses. p^1^ represents the difference at week 12 versus baseline; p2 represents the difference at week 24 versus baseline; and p^3^ represents the difference at week 52 versus baseline. This analysis is shown univariably. Statistically significant values are shown in bold.

BMI, body mass index; CDAI, Clinical Disease Activity Index; CRP, C reactive protein; DAS28, Disease Activation Score using 28 joints; DMARD, Disease-modifying antirheumatic drugs; ESR, erythrocyte sedimentation rate; HAQ, Health Assessment Questionnaire; SDAI, Simple Disease Activity Index.

### 3.2 Changes in the basal activity of complement system pathways during one year of follow-up

**Figure 1:**
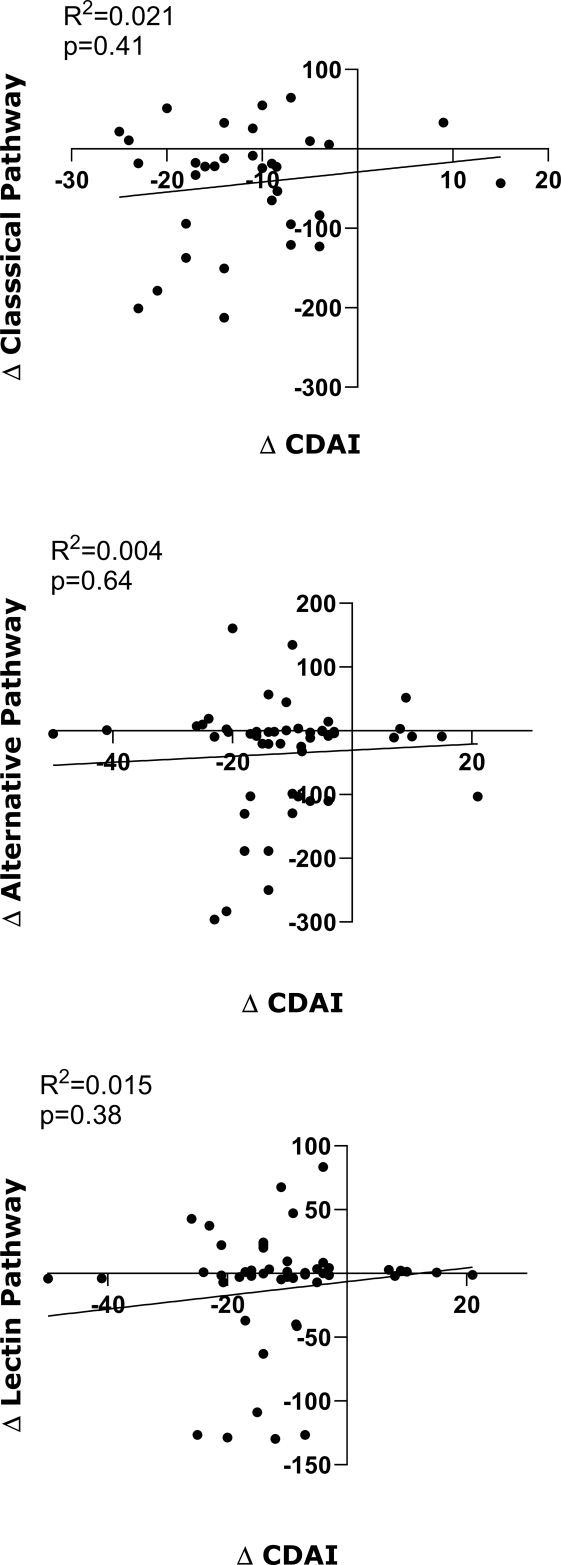
Correlation of variations in baseline values with respect to those at 12, 24 and 54 weeks of TCZ treatment. Each dot represents the difference between the basal value and each of those time points of disease activity (ΔCDAI), and the functional activity of the Classical pathway (ΔClassical pathway), the Alternative pathway (ΔAlternative pathway) and the Lectin pathway (ΔLectin pathway) of the complement. The x-axis represents variations in CDAI, and the y-axis represents variations in functional activity of each of the complement pathways. Correlation values and statistical significance are shown in each figure.

**Table 3** shows the univariable and multivariable analysis of the basal functional activity variation of the three complement pathways over one year of treatment with TCZ in RA patients. The classical pathway exhibited significant decreases at weeks 12, 24, and 52 compared to baseline. These changes remained significant in the multivariable adjustment, including disease activity as assessed by the CDAI index, which excludes phase reactants and has been proposed as the optimal index for evaluating RA activity (22), particularly in patients undergoing anti-IL6 treatment. The alternative pathway showed significant decreases at weeks 12 and 24, but not at 52; these changes also remained significant after adjusting for disease activity. Conversely, the functional test for the lectin pathway showed no changes throughout the follow-up period (**Table 3**). In accordance with the multivariate analysis, no correlation was identified between variations in disease activity and changes in the functional activity of the three complement pathways when these were analyzed over the course of TCZ treatment (**Figure 1**).

**Table 3:** Multivariable analysis of changes in complement system pathways functional assay values during follow-up.

Classical, %	Univariable Mean difference (95% CI)		Multivariable Mean difference (95% CI)	
		p-value		p-value
Basal	124 ± 52			
Week 12	**-32 (-58-(-6))**	**0.016**	**-36 (-66-(-5))**	**0.022**
Week 24	**-40 (-67-(-14))**	**0.003**	**-44 (-74-(-14))**	**0.004**
Week 52	**-33 (-59-(-6))**	**0.015**	**-37 (-68-(-6))**	**0.020**
Alternative, %				
Basal	29 (22-123)			
Week 12	**-39 (-71-(-8))**	**0.014**	**-47 (-85-(-10)**	**0.013**
Week 24	**-46 (-79-(-14))**	**0.005**	**-56 (-94-(-17)**	**0.005**
Week 52	-23 (-55-10)	0.17	-28 (-69-12)	0.17
Lectin, %				
Basal	25 (22-64)			
Week 12	-27 (-66-13)	0.19	-20 (-64-25)	0.38
Week 24	-19 (-60-22)	0.36	-24 (-70-23)	0.32
Week 52	-22 (-63-19)	0.30	-28 (-75-20)	0.25

Multivariable analysis is adjusted for CDAI (Clinical Disease Activity Index).

Differences are shown using the basal value of the corresponding pathway as the reference. Therefore, mean differences represent the differences at weeks 12, 24, and 52 compared to baseline, using mixed models for continuous responses. These analyses were performed using linear mixed models. Statistically significant values are shown in bold.

## 4 Discussion

Our study is the first in the literature to analyze the effects of IL-6 inhibition on the complement system through a functional characterization of its three pathways. Our results indicate that TCZ decreases the values of the functional tests of the classical and alternative pathways, but not the lectin cascade in RA patients. Notably, this effect of TCZ occurs independently of the drug’s beneficial impact on RA disease activity.

RA is a chronic autoimmune disease that causes articular and extra-articular damage due to tissue infiltration by leukocytes and prolonged systemic inflammation driven by proinflammatory cytokines, including IL-6 (23). IL-6 is a pleotropic cytokine that plays a key role in many aspects of the immune response, including acute-phase response (7–11). The clinical benefit of IL-6 inhibition by TCZ in RA patients (1, 24) has been attributed, in part, to its favorable impact on bone and cartilage turnover, inflammation, and joint damage. This is achieved through its effects on chondrocytes, osteoclasts, macrophages, fibroblasts, T and B cell differentiation, and its reduction of the acute phase response (25). Other mechanisms by which TCZ reduces disease activity in RA patients, such as modulation of complement system activity, have only been partially explored in the existing literature. For example, in a previous report, serum levels of the complement components C3 and C4 were retrospectively assessed in 19 consecutive RA patients eligible for TCZ treatment (26). C3 and C4 were found to decrease as early as 4 weeks after the first TCZ infusion and this effect was maintained after 12 months of therapy. A discrete proportion of patients (38.46% and 30.76% for C3 and C4, respectively) had reduced serum complement levels, raising the possibility that complement consumption was at work. In this regard, after 12 months of therapy, 69% and 56% of patients had abnormally low C3 and C4 serum levels, respectively. The authors concluded in this report (26) that complement reduction is not a short-term effect of IL-6 signaling inhibition: patients still on TCZ therapy after years of continuous drug exposure continue to have significantly reduced or subnormal serum C3 and C4 levels. Our findings are consistent with this report, as we believe that the observed decrease in complement functional assays found in our study most likely reflects the downregulation of complement protein synthesis by IL-6 inhibition rather than its consumption by activation. However, it is important to note that none of the patients in the study displayed signs or symptoms suggestive of immune complex disease, such as purpura, glomerulonephritis, flare-ups of arthritis, and/or fever (26). Similar results were found in a report of 108 patients with RA followed up during a mean period of 4.9 years (range, 1–14 years) (27). In this study, 30% of the patients developed low C4 levels and 21% also had low levels of C3. Low C3/C4 levels correlated with prolonged TCZ treatment retention time and effectiveness. This was also the case in the double-blind, placebo-controlled, randomized trial, which examined the effects of TCZ in patients with RA (OPTION study) (24). In this study, mean levels of C3 and C4 proteins decreased from baseline in the TCZ groups but remained within the normal range during follow-up. In SLE, IL-6 blockade has produced effects on complement proteins similar to those observed in RA but has not demonstrated clinically relevant effects on disease control. Both in a small study of 16 patients (28), and in a placebo-controlled phase II clinical trial of 186 patients (29) with mild to moderate SLE, TCZ (28) and another IL-6 monoclonal antibody (29) produced a significant dose-related reduction in basal complement levels. All these studies have focused on determining individual complement proteins belonging mainly to the classical pathway, but none of them have analyzed complement pathways from a functional perspective, as we have done in our study. This new approach allows for a more comprehensive analysis of the entire complement system, rather than being limited to the analysis of some of its individual components.

Remarkably, in our study TCZ’s influence on the complement system activity was found to be significant for the classical and alternative routes, but not the for the lectin cascade. It is known that lectin pathway deficiency is a prevalent condition, affecting approximately 5–30% of the general population, which highlights the redundancy of the immune system (30). RA patients most likely also exhibit this deficiency, which would account for the lack of TCZ’s impact on the lectin pathway. Moreover, it has been observed that CRP levels drop quickly after TCZ treatment has been initiated (24). CRP is an acute-phase serum protein and a mediator of innate immunity that binds to microbial polysaccharides and ligands exposed on damaged cells. CRP initiates the classical pathway by activating C1q (9). The effect that TCZ has on CRP probably influences initiation of the classical pathway, eventually causing some distortion in the activity of both the classical and alternative cascades. Besides, it is well established that IL-6 is the primary inducer of hepatic acute phase proteins. Furthermore, the liver, predominantly hepatocytes, is responsible for the biosynthesis of approximately 80-90% of plasma complement components and expresses a diverse array of complement receptors (31). We postulate that the effect of IL-6 inhibition on liver metabolism, particularly on acute phase reactants, is likely the reason why only the classical and alternative complement pathways were affected by the drug.

Serum CH50 levels are usually normal or elevated in RA patients compared to controls (17) and have been reported to be even higher in RA patients without severe extra-articular manifestations (16). Disease activity is thought to increase these functional tests due to the acute-phase response present in the disease. In our study, we observed a decrease in the values of the functional tests of the classical and alternative pathways after TCZ treatment in RA patients. The decrease observed in these functional tests on the complement most likely reflects the downregulation of complement system protein synthesis by IL-6 inhibition, rather than its consumption by activation.

In our study, changes in complement cascade activity levels over time were adjusted for variations in disease activity using the CDAI score. This composite index does not include measures of acute phase reactants and is considered the optimal index for assessing RA disease activity (22), particularly in patients receiving anti-IL-6 treatment (32). Notably, after adjusting for CDAI, the changes observed in the univariable analysis remained significant. With respect to this question, no correlation was identified between variations in disease activity and changes in functional activity in any of the three complement pathways analyzed at any time throughout the follow-up period. This suggests that the changes TCZ exerts on complement pathway activity may be a direct effect of the drug on complement protein synthesis rather than a consequence of changes in disease activity.

The main strength of our study is that it includes systematic clinical and biochemical evaluations of 27 RA patients undergoing systemic IL-6 inhibition during 1-year of follow-up using multivariable linear mixed models for the analysis, allowing us to examine the long-term effects of TCZ on complement pathways. Although our aim was to investigate the effects of TCZ on functional tests of complement pathways, we acknowledge the limitation of not having directly measured individual elements of the complement pathways, determinations that have already been partially studied in previous work. However, the complement system is a complex network of molecules, regulators, and inhibitors, for which no gold standard test exists. Our hypothesis of measuring the three pathways functionally, while limited, provides a broad overview of how complement pathways function may change following TCZ treatment. Furthermore, it is known that the activity of the complement system may differ between males and females (33–35). The fact that our study was conducted only in females prevents extrapolation of the data to male patients. Similar studies in male subjects will be necessary to determine whether the effect of TCZ on the complement system is equivalent to that shown in our results in females.

In conclusion, TCZ has demonstrated to reduce the values of both the classical and alternative complement pathways functional tests in RA patients, regardless of changes in disease activity. Our study supports the concept that the anti-inflammatory effect of TCZ in RA may be mediated, in part, by its effect on the complement system. This finding underscores the complex interplay between IL-6 inhibition and complement activation in the pathogenesis of RA.

## Data Availability

The raw data supporting the conclusions of this article will be made available by the authors, without undue reservation.
